# Call Me Maybe… A Simulation Based Curriculum for Telephone Triage Education in a Pediatric Residency

**DOI:** 10.3389/fped.2020.00283

**Published:** 2020-06-23

**Authors:** Joel S. Blumberg, Michelle Barajaz, Danielle Roberts, Cody Clary, Shelley Kumar

**Affiliations:** ^1^Department of Clinical Sciences, University of Houston College of Medicine, Houston, TX, United States; ^2^Department of Pediatrics, Baylor College of Medicine, San Antonio, TX, United States; ^3^Section of Neonatology, Department of Pediatrics, University of Colorado School of Medicine, Aurora, CO, United States; ^4^Center for Research, Innovation and Scholarship in Medical Education, Baylor College of Medicine, Texas Children's Hospital, Houston, TX, United States

**Keywords:** telephone, triage, simulation, pediatric, education

## Abstract

Pediatrician communication with caregivers by phone has traditionally made a significant impact on patient care but remains a source of medical liability. Despite its importance, few publications exist regarding the education of pediatric residents on telephone triage. Our study involved the development of an educational curriculum aimed at expanding the pediatric resident skill set in telephone triage. Our method of curriculum development is based on Kolb's experiential learning theory. We utilized a combination of resource familiarization, didactic education, and simulation in the building of knowledge through reflection upon concrete experience, generalization of knowledge gained, and application of this new knowledge. We developed a 30-min PowerPoint presentation in which instructors reviewed the basic tenets of telephone triage. In the pilot study, residents were divided into two groups—a didactic-first group and a simulation-first group. Their performance was monitored during two scripted, symptom based “parent” phone call simulations. The didactic-first group received the PowerPoint didactic prior to the simulation, and the simulation-first group received the didactic after the simulation. A comparison of resident evaluations by faculty and self-documented confidence level revealed statistically significant higher evaluation scores in the didactic-first group, and an overall improvement in resident confidence with telephone triage. We conclude that this educational curriculum may improve pediatric resident performance in telephone triage.

## Introduction

In addition to their clinical work in the inpatient and outpatient settings, pediatricians have traditionally been called upon to speak with caregivers by phone regarding chief complaints on pediatric patients who are not present for a physical examination. These conversations serve as a form of triage to establish which patients require immediate medical intervention vs. those who may be safely cared for at home. Pediatric telephone triage can have a significant impact on the health and well-being of patients, but can also significant source of medical liability.

A review of the literature reveals very few past studies assessing resident telephone triage skills, resident confidence level, and educational models for telephone triage. The studies that are published in this area date to the late 1990s (1). One study indicated that fewer than half (45%) of programs surveyed offered specific training in telephone triage, and that of those programs who offered resident training, the most common instructional method was by formal lecture rather than direct experience with simulation (2). Simulated telephone triage has been explored previously as a method to teach professionalism and communication skills among an inter-professional team using “mock page” scenarios (3). However, no other published curriculum was identified evaluating the effectiveness of skill development and confidence level using telephone triage simulation in a pediatric residency. Our study sought to fill this gap.

Kolb's experiential learning theory describes the building of knowledge via reflection upon concrete experience, generalization of knowledge gained, and application of this knowledge (4, 5). We utilized this structure to develop an educational strategy aimed at expanding the pediatric resident skill set in telephone triage. We hypothesized that pediatric resident physicians would benefit from training in telephone triage. The aim of our study was to assess the need for such training, create an educational activity to provide such training if needed, and to demonstrate measurable improvement in the provision of telephone triage following participation in any activity designed.

## Materials and Methods

**Figure 1:**
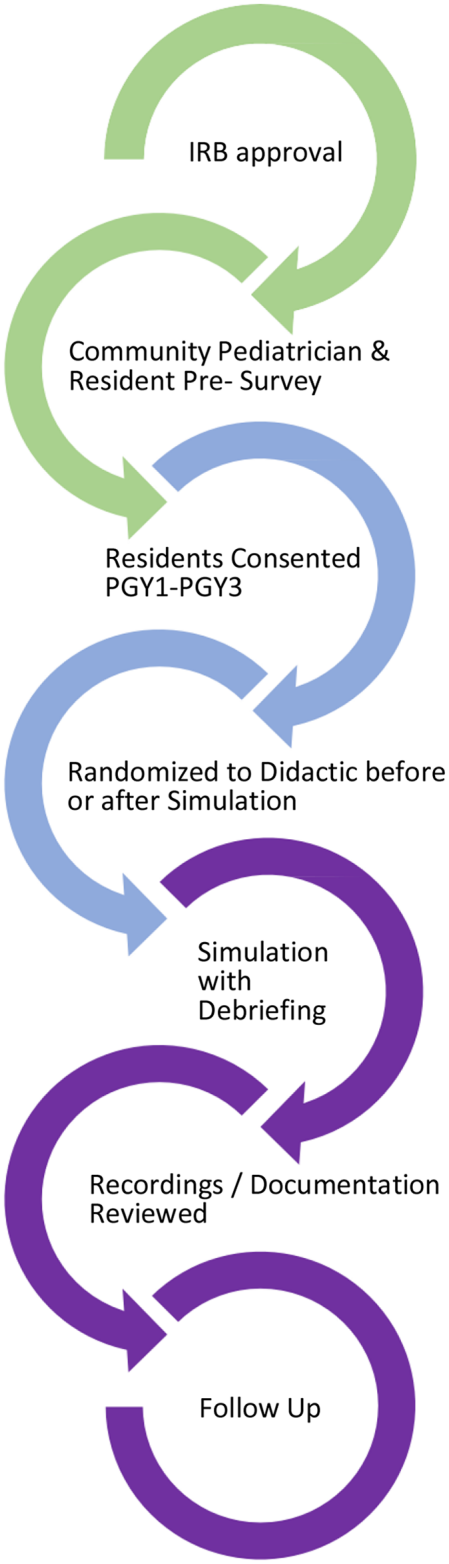
Study methodology flow diagram.

Our study was performed with participants from the Baylor College of Medicine—San Antonio pediatric residency program. We utilized the simulation lab at Christus Health's Children's Hospital of San Antonio for the simulations. We began by obtaining approval from the Baylor College of Medicine and Christus Health Institutional Review Boards (IRBs) (Figure 1). A needs assessment survey was emailed to 27 community and faculty pediatricians in order to determine the perceived need for telephone triage training during residency, the best timing for this training to occur, high yield chief complaints, and any additional comments from providers (Appendix A). A needs assessment survey was also provided to residents to evaluate confidence level with telephone triage, familiarity with resources available to help with telephone triage, level of interest in a formal curriculum during residency, and current skill set in triage scenarios (Appendix B).

Residents recruited for the study were divided into two groups. Randomization was achieved utilizing computer generated random numbers assigned to an alphabetical list of participating residents of each post-graduate year. Following this randomization, two of the residents were allowed to switch groups due to a scheduling conflict. In this manner, groups were formed maintaining a balance between post-graduate year and group size. Of the subjects enrolled, 12 were from the intern year, 8 were second year residents, and 9 were third year residents.

Residents were randomized as noted to a didactic-before simulation group (didactic-first) or a didactic-after simulation (simulation-first) group. Residents were informed of their group placement by receiving a slip of paper with the letter (A) or (B) on it: (A) corresponding to the didactic-first and (B) corresponding to the simulation-first grouping. They were also assigned a subject number that would be recorded during the scenarios to maintain anonymity. Group (A) had 15 participants and group (B) had 14 participants. Residents were blinded to whether or not they would be receiving the didactic before or after the simulations. We provided Barton Schmitt's *Pediatric Telephone Protocols*: 16th edition (6) as well as the Tips for Successful Telephone Triage pocket card (Appendix C) to be available prior to their follow-up simulated phone calls. All residents were gathered for 3 h during a Friday didactic session already scheduled for residency education. Group (A) received a 30-min interactive PowerPoint didactic covering the basics of telephone triage. Scenarios different from those provided through simulation were presented at the end of the lecture for residents to practice the skills demonstrated in the PowerPoint. Group (B) went to the simulation center at the Children's Hospital of San Antonio.

Each resident individually entered a simulation room from which the audio portion of their telephone conversation could be recorded remotely. A phone was provided and a sign indicated the telephone number to call for scenario 1 (Abdominal Pain script) and scenario 2 (Headache script). A faculty physician was waiting in a separate room to receive the phone call and begin the simulation. Each faculty member was provided a script and asked to play the role of caregiver. The faculty became familiar with the script and were then instructed to provide information to the resident only when asked. They were allowed to improvise as necessary when asked a question not addressed in the script. Scenarios lasted 10–15 min on average unless the resident did not request a call back number at the beginning of the conversation at which time the phone call was disconnected. Residents were expected to illicit the history and physical exam findings using the parent as a remote examiner. The resident was then expected to provide appropriate disposition for the patient. A precise and accurate diagnosis or disposition did not factor into the evaluation process. The simulation concluded with resident documentation of the scenario on a pre-printed template. When all group B residents had completed the scenarios, a large group debriefing was held to gather resident feedback on the activity.

Approximately 1 month later, during another 3 h Friday didactic session, group A performed the simulation while group B received the PowerPoint presentation. All variables remained unchanged between the two simulation days. Residents were evaluated using a standardized rubric for both scenarios (Appendix D). The assessments were performed by two blinded physicians, one of whom listened to the recordings and one of whom reviewed the written documentation, with performance based on the rubric. A resident post-survey was administered to provide feedback regarding the simulation and post-intervention knowledge on telephone triage (Appendix E). After 2 months, both groups returned to the simulation center to complete a third scenario, which assessed the retention of telephone triage skills.

### Data Analysis

A total score was calculated by summing the scores on the graded simulation performance as well as the written documents for each scenario. An independent-samples *t*-test was conducted to compare this total score between didactic-first and simulation-first for each scenario. A Wilcoxon signed rank test was also conducted to compare the difference in mean scores between the first two scenarios with the third scenario within each group, to see if prior simulation improved performance. Pre- and post-unlinked confidence medians were compared using Wilcoxon rank sum test. Statistical analysis was performed by using SAS 9.4 (SAS Institute, Cary, NC). The statistical tests were two-sided, and a *p*-value of <0.05 was used to indicate statistical significance. No power calculation was performed.

## Results

Fifteen of the 27 pediatricians (56%) and 25 of the 30 residents (83%) queried with the study needs assessment responded. The feedback we received from our needs assessments strongly supported our perceived need for telephone triage instruction, leading us to proceed with the development of the educational component. 73.3% of the responding pediatricians stated that it was “extremely important” to train the residents in proper telephone triage. Forty percent stated that if utilized, a curriculum in telephone triage would be best implemented in the second year whereas 33.3% stated that the best time of implementation would be just prior to finishing residency in the third year. The three most important triage skills stated were following:

- Providing appropriate interim care and escalated instructions (84.6%)
- Assessing the patients understanding of the advice given (53.9%)
- Quickly assessing for emergency conditions (53.9%)

**Figure 2:**
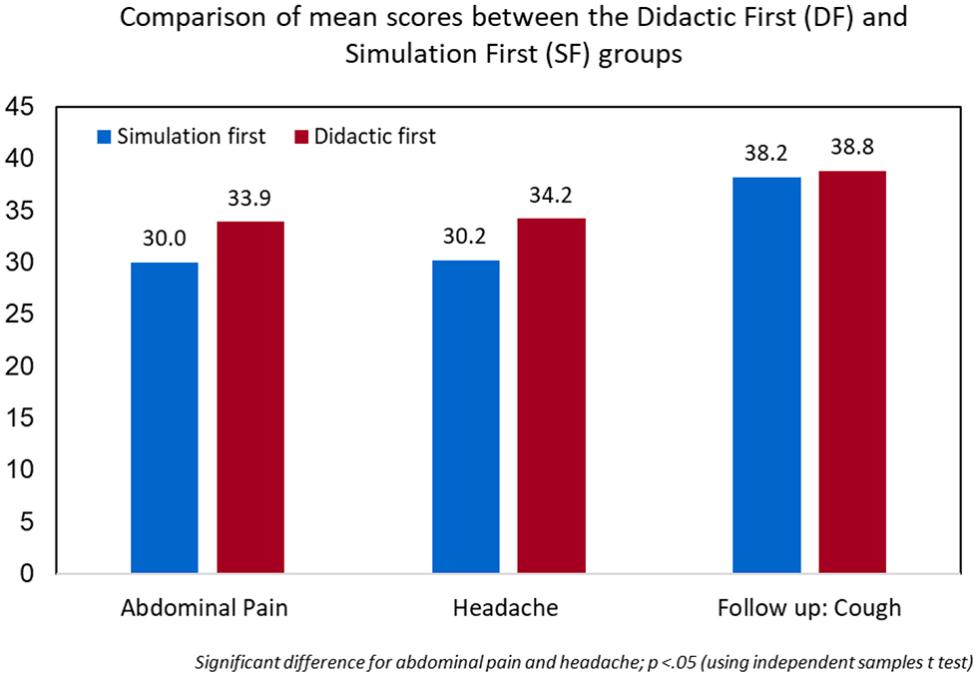
Independent *t*-test showed significantly higher rubric score for residents performing the simulation after didactic (*p* = 0.029, 0.009, 0.656).

Our study enrolled 29 pediatric residents. Fourteen residents were randomized to receive the simulation first. The remaining 15 residents were randomized to receive the didactic first. The *t*-test comparison showed that for the chief complaint of abdominal pain scenario, mean rubric score was higher for the didactic-first group (33.9) than the simulation-first group (30.0), *p* = 0.029. For the chief complaint of headache scenario, the mean rubric score of didactic-first (34.2) was also statistically significantly higher than for the simulation-first (30.2), *p* = 0.009. For the follow up cough scenario performed ~2 months after initial testing, no statistically significant difference was shown between the mean scores of the two groups (simulation-first = 38.2 vs. didactic-first = 38.8, *p* = 0.656). An independent *t*-test showed significantly higher rubric score for residents performing the simulation after didactic (*p* = 0.029, 0.009, 0.656) (Figure 2). Though we were limited by sample size to see statistical differences in performance between PGY years, a descriptive table has been added in the Supplementary Material (Table 1).

**Table 1:** Simulation scores of the simulation-first and didactic-first groups by post-graduate year of the residents.

**Group**	**Resident year (*n*)**	**Total scores for each scenario**		
		**Abdominal pain**	**Headache**	**Cough**
Simulation-first	PGY1 (7)	29.9	27.7	36.8
	PGY2 (3)	28.7	32.7	39.3
	PGY3 (4)	31.3	33.0	40.5
Didactic-first	PGY1 (5)	33.8	34.0	33.0
	PGY2 (5)	33.2	33.2	40.5
	PGY3 (5)	33.6	37.2	40.0

A Wilcoxon signed rank test indicated that scores on the cough scenario were higher than headache for simulation-first (*p* = 0.001) as well as didactic-first (*p* = 0.016). Also, the scores on cough were higher than abdominal pain for simulation-first (*p* = 0.001) as well as didactic-first (*p* = 0.008) group.

**Figure 3:**
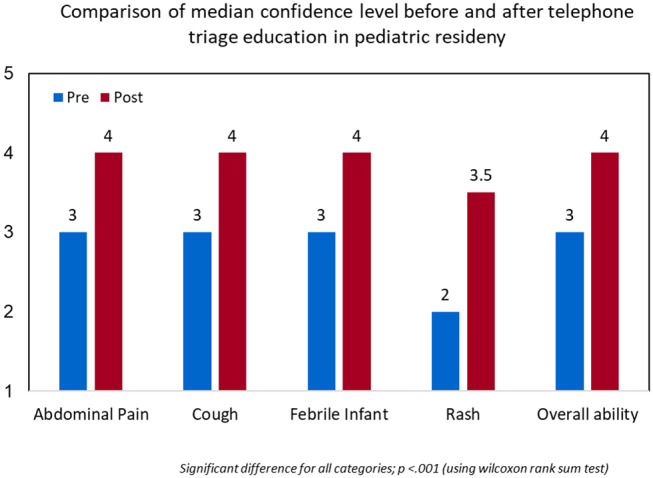
Resident confidence increased following training, and translated to other scenarios.

Using the Wilcoxon rank sum test, resident median confidence level in addressing common chief complaints during telephone triage was shown to be significantly higher after the simulation exercises and reached statistical significance in all categories (Figure 3). The median score with interquartile range for each complaint is provided (Table 2).

**Table 2:** Resident median confidence level in addressing common chief complaints during telephone triage.

**Chief complaint**	**Pre**	**Post**	p^*^
Abdominal pain	3 (2–3)	4 (4–5)	<0.0001
Cough	3 (3–4)	4(4–5)	<0.0001
Febrile infant	3(3–4)	4 (4–5)	<0.0001
Rash	2 (2–3)	3.5 (2.5–5)	0.0002
Overall	3 (2–3)	4 (4–4)	<0.0001

* *Due to unlinked pre-post data, Wilcoxon rank sum test was used to calculated p-values*.

Additionally, residents had favorable responses to the training activity and subjectively determined that it was a beneficial experience based on free-text comments provided through post-event survey. Sample comments include, “The simulation was like real-life scenarios,” “I liked the different methods of learning,” “I liked the reference cards and learning the basics,” and “Good information about something we don't get a lot of experience with.”

## Discussion

Following a needs assessment which demonstrated a perceived need for resident training in telephone triage, our study sought to develop an educational strategy aimed at expanding the pediatric resident skill set in this area. We accomplished this goal with the use of Kolb's experiential learning theory and the Kern approach to curriculum development in the creation of a novel simulation based telephone triage curriculum for pediatric residents (7). Overall, the curriculum was shown to be effective at enhancing the resident skill set and subjective confidence level in telephone triage. Providing a didactic component prior to simulation helped improve resident performance on initial evaluation and both groups demonstrated retention of telephone triage skills upon follow up and performed equally well on the final scenario. An added benefit to the project was resident perceived usefulness of the activity. Resident feedback was overall positive, and the activity was felt to be useful in preparation for their future careers as independently practicing physicians. This curriculum is likely generalizable to other pediatric and possibly additional health professions training programs. Minimal resource utilization and adaptable case scenarios indicate high feasibility for implementation in other healthcare-related educational environments.

Despite many positive aspects, our study does face several challenges and limitations. The first challenge encountered occurred during the simulations. As a small residency program, our residents and faculty work very closely together; therefore, it is likely that some faculty and resident identities were inadvertently revealed during the simulation through recognition of voices by phone. Although residents did not indicate this as a concern on follow up surveys, it was not directly addressed during the debriefing session or on post-survey follow up. This limitation could be addressed by involving individuals who are not in direct communication with residents frequently. It may also be beneficial to utilize individuals who are not medically trained to play the role of parent. Faculty were asked to comply with the role outlined in the script provided, but it is possible that their medical training altered their improvised responses. In addition, although the calls were assessed by single individuals (audio or written), the rubric used was not independently tested for validity or reliability. There were also, due to time and manpower constraints, a limited number of scenarios assessed. The final limitation recognized in our study is its small sample size. This was secondary to the nature of a small residency program. The statistical significance of our primary outcomes could be altered by an increase in the number of study participants, as well as a more complete randomization. The challenges of implementing this curriculum will likely increase as the number of participants increase due to the need for more resources required to carry out the simulations.

## Conclusion

It is critically important for pediatric physicians to be able to obtain the necessary information, assess clinical condition, and formulate a disposition appropriate to the patient case through telephone triage. Despite the limitations of our study, experiencing telephone triage calls in a real-time, low-risk setting, such as a simulation lab seems to be an effective learning strategy for pediatric residents.

## Data Availability Statement

The data supporting the conclusions of this article will be made available by the authors, without undue reservation, to any qualified researcher.

## Ethics Statement

The studies involving human participants were reviewed and approved by Baylor College of Medicine and Christus Healthcare. The patients/participants provided their written informed consent to participate in this study.

## Author Contributions

JB, MB, DR, and CC participated in the design and implementation of the study. JB and DR created the didactic portion of the training, which was presented to the residents by DR and CC. Statistical analysis was provided by SK. All authors contributed to the article and approved the submitted version.

## Conflict of Interest

The authors declare that the research was conducted in the absence of any commercial or financial relationships that could be construed as a potential conflict of interest.
