# Supplementary material for: Call Me Maybe… A Simulation Based Curriculum for Telephone Triage Education in a Pediatric Residency
Source: Front Pediatr. 2020 Jun 23;8:283. doi: 10.3389/fped.2020.00283 (PMC7324759; doi:10.3389/fped.2020.00283)
Supplement: Supplementary file 1 [file Data_Sheet_1.docx]

Supplementary Material

**Appendix A**

Practicing Pediatrician Survey of Resident Needs – Telephone Triage Curriculum

This brief survey is designed to help us identify what skills and topics you feel are most important to teach our residents to prepare them to successfully manage telephone triage/after-hours patient phone calls. It should take less than 5 minutes to complete. Your input is invaluable and greatly appreciated.

Demographic information: Practice setting

1. On a scale of 1-5, how important do you feel it is to train our residents in proper telephone triage, where: 1 = It needs no training, it is an innate skill, and 5 = They won’t do it right unless we teach them how.

1 2 3 4 5

1. Rank in order of importance the following phone triage tasks:

___ Obtaining complete demographic date (e.g., patient’s name, DOB, caller’s name and

relation to patient, call-back phone number, patient’s PCP).

___ Getting complete symptoms.

___ Arriving at the correct diagnosis.

___ Choosing the correct disposition (e.g., send patient to ED, call for next day appointment, or

manage safely at home).

___ Obtaining Past Medical History and current medications.

___ Assessing parent’s understanding of advice given.

___ Arranging for follow-up by appointment or phone call.

___ Complete documentation of the phone call.

1. When do you feel it is best to train our residents in proper telephone triage:

___ The sooner, the better! 1st year

___ Wait until they’ve rotated through Ambulatory Peds Clinic at least once. 2nd year

___ Not until just before they’re going to start taking After-Hours Phone Call. 3rd year

___ Start their 1st year, with refresher training the 2nd and 3rd years.

1. Which patient symptoms are most important to cover in this training? Please rank the 5 most important.

___ Febrile infant, 0-3 months old

___ Febrile infant, >3 months

___ Febrile child, 12 months and older

___ Fussy infant/irritable child

___ GI symptoms (e.g., vomiting, diarrhea, abdominal pain)

___ Respiratory symptoms (e.g., cough, wheezing, stridor)

___ Rashes

___ Injuries and foreign bodies

___ Non-injury Ortho symptoms (e.g., limp, joint swelling)

___ Other Neuro symptoms (e.g., headache, seizures, weakness)

___ Other, please list: _____________________________

_____________________________

_____________________________

Are there additional skills you feel we should address? Please list.

**Appendix B**

Resident Needs Assessment Survey – Telephone Triage Curriculum

This brief survey is designed to assess your level of experience, views, and needs regarding a Telephone Triage Curriculum at the Children’s Hospital of San Antonio. It will take approximately 5 minutes to complete. For the following questions, the responses are: 1- not confident, 2-somewhat confident, 3- confident, 4- very confident, 5- extremely confident

1. Rate your confidence in your overall ability to field calls about sick children.
2. 2 3 4 5
3. Rate your confidence managing phone calls relating to the following chief complaints:

Febrile infant 1 2 3 4 5

Vomiting/diarrhea 1 2 3 4 5

Cough 1 2 3 4 5

Headache 1 2 3 4 5

Abdominal Pain 1 2 3 4 5

Rash 1 2 3 4 5

1. Do you know what resources you can use to help you decide if a patient can wait to be seen or needs to go to the ER? Yes No Unsure
2. How confident are you in dealing with the following parents:

a. Angry 1 2 3 4 5

b. Incoherent 1 2 3 4 5

c. Do not seem to comprehend given instructions 1 2 3 4 5

1. On a scale of 1-5, how important is it for residents to incorporate telephone triage into their medical training (1- not important; 5 extremely important)?

1 2 3 4 5

1. Rank how well the following teaching methods match your learning style

(1- no match, 2- somewhat match, 3- match, 4- great match, 5- perfect match):

a. Online interactive module with incorporated questions 1 2 3 4 5

b. Phone call simulations with actors/volunteers 1 2 3 4 5

c. Power Point presentation with assigned textbook reading 1 2 3 4 5

1. What is the first thing to ask when receiving a phone call from a caregiver?

a. Chief Complaint

b. Name and call back number

c. Mental status of patient

d. How their day is going

1. Do you have specific scenarios that you would like some training on how to manage?
2. What do you foresee your biggest struggle being with fielding calls?

**Appendix C**

| **Tips for Successful Telephone Triage** |
| --- |
|  |
| **Gather appropriate initial information:**  □ Call back number □ Patient name □ DOB □ Caller □ PCP |
| **Assess for medical confounders:**  □ Chronic conditions □ Medications □ Sick contacts □ Recent illness □ Trauma |
| **Assess for emergency conditions such as:**  □ Dehydration/shock □ AMS □ Resp distress □ Acute abdomen □ Life-threatening infection/injury |
| **Utilize remote physical exam skills when needed such as:**  □Instruct caregiver in counting HR or RR, describing rash characteristics, localizing or identifying pain, or performing simple physical exam maneuvers; listen over the phone, etc. |
| **Assess for social confounders such as:**  □ Transportation issues □ Risk for abuse/neglect □ Ability to pay for medical necessities □ Concerns for care environment (travel, available general necessities, parental support) |
| **Provide appropriate disposition:**  □Quickly and appropriately decide if the child needs emergency intervention and whether 911 or private transportation is appropriate.  □Determine appropriate length of time before non-emergent medical care is received. □Gain caregiver buy-in for disposition |
| **Provide interim care/escalation instructions such as:**  □ Diet □ Medications □ Other supportive therapies □ Concerning signs □ Actions to take if these signs occur (when and where to go) |
| **Utilize appropriate interpersonal and communication skills:**  □ Uses appropriate phone etiquette □ Avoid medical jargon □ Assess understanding of Instructions □ Communicate empathy and support |
| **Document call appropriately:**   - Note date, time, patient, and caller □ Reason for call □ Confounding information □ History and physical items that support disposition - Recommendations □ Caregiver agreement □ Route documentation appropriately |

**Appendix D**

| **Pediatric Telephone Triage Simulation Scoring Rubric** | Does poorly; many important pieces missed that could affect outcome, or many unnecessary elements |  | Some missed elements, which most likely will not affect outcome, or some unnecessary elements |  | Does  extremely  Well; no missed elements, minimal unnecessary elements |
| --- | --- | --- | --- | --- | --- |
|  | 1 | 2 | 3 | 4 | 5 |
| **Gather appropriate initial information:**  □ Call back number □ Patient name □ DOB □ Caller □ PCP |  |  |  |  |  |
| **Assess for medical confounders:**  □ Chronic conditions □ Medications □ Sick contacts □ Recent illness □ Trauma |  |  |  |  |  |
| **Assess for emergency conditions such as:**  □ Dehydration/shock □ AMS □ Resp distress □ Acute abdomen □ Life-threatening infection/injury |  |  |  |  |  |
| **Utilize remote physical exam skills when needed such as:**  □Instruct caregiver in counting HR or RR, describing rash characteristics, localizing or identifying pain, or performing simple physical exam maneuvers; listen over the phone, etc. |  |  |  |  |  |
| **Assess for social confounders such as:**  □ Transportation issues □ Risk for abuse/neglect □ Ability to pay for medical necessities □ Concerns for care environment (travel, available general necessities, parental support) |  |  |  |  |  |
| **Provide appropriate disposition:**  □Quickly and appropriately decide if the child needs emergency intervention and whether 911 or private transportation is appropriate.  □Determine appropriate length of time before non-emergent medical care is received. □Gain caregiver buy-in for disposition |  |  |  |  |  |
| **Provide interim care/escalation instructions such as:**  □ Diet □ Medications □ Other supportive therapies □ Concerning signs □ Actions to take if these signs occur (when and where to go) |  |  |  |  |  |
| **Utilize appropriate interpersonal and communication skills:**  □ Uses appropriate phone etiquette □ Avoid medical jargon □ Assess understanding of Instructions □ Communicate empathy and support |  |  |  |  |  |
| **Document call appropriately:**   - Note date, time, patient, and caller □ Reason for call □ Confounding information □ History and physical items that support disposition - Recommendations □ Caregiver agreement □ Route documentation appropriately |  |  |  |  |  |
| **Total Score:** |  |  |  |  |  |

**Appendix E**

**Resident Post-Activity Survey – Telephone Triage Curriculum**

This brief survey is designed to assess your thoughts and test your knowledge about triaging patient phone calls after participating in the Telephone Triage Curriculum at the Children’s Hospital of San Antonio. It will take approximately 5 minutes to complete.

For the following questions, the responses are: 1- not confident, 2-somewhat confident, 3- confident, 4- very confident, 5- completely confident

1. Rate your confidence in your overall ability to field calls about sick children. 1 2 3 4 5
2. Rate your confidence managing phone calls relating to the following chief complaints: (based on practicing pediatrician survey)
   1. Febrile patient 1 2 3 4 5
   2. Cough 1 2 3 4 5
   3. Rash 1 2 3 4 5
   4. Abdominal Pain 1 2 3 4 5
3. Do you know what resources you can use to help you decide if a patient can wait to be seen or needs to go to the ER? Yes No Unsure
4. What is the first thing to ask when receiving a phone call from a caregiver?
   1. Chief Complaint
   2. Name and call back number
   3. Mental status of patient
   4. How their day is going
5. Rank the **didactic (PowerPoint)** portion of the activity in the following areas from 1 to 5 (1- completely disagree, 2- disagree, 3- somewhat agree, 4- agree, 5- completely agree):
   1. It has helped me acquire telephone triage skills 1 2 3 4 5
   2. It applies to real life situations 1 2 3 4 5
6. Rank the **simulation (mock calls)** portion of the activity in the following areas from 1 to 5 (1- completely disagree, 2- disagree, 3- somewhat agree, 4- agree, 5- completely agree):
   1. It has helped me acquire telephone triage skills 1 2 3 4 5
   2. It applies to real life situations 1 2 3 4 5
7. What is the likelihood you will use these skills in patient care? (1- unlikely, 2-somewhat likely, 3- likely, 4- very likely, 5- completely likely):

1 2 3 4 5

1. What did you like about this curriculum?
2. What did you find less helpful about the curriculum?
3. What additional comments do you have?
